# Pokémon GO and psychological distress, physical complaints, and work performance among adult workers: a retrospective cohort study

**DOI:** 10.1038/s41598-017-11176-2

**Published:** 2017-09-07

**Authors:** Kazuhiro Watanabe, Norito Kawakami, Kotaro Imamura, Akiomi Inoue, Akihito Shimazu, Toru Yoshikawa, Hisanori Hiro, Yumi Asai, Yuko Odagiri, Etsuko Yoshikawa, Akizumi Tsutsumi

**Affiliations:** 1 Department of Mental Health, Graduate School of Medicine, The University of Tokyo, Tokyo, 113-0033 Japan; 2 The Japan Society for the Promotion of Science, Tokyo, 102-0083 Japan; 3 Department of Public Health, Kitasato University School of Medicine, Kanagawa, 252-0374 Japan; 4 Center for Human and Social Sciences, Kitasato University College of Liberal Arts and Sciences, Kanagawa, 252-0373 Japan; 5 National Institute of Occupational Safety and Health, Japan, Kanagawa, 214-8585 Japan; 6 Department of Mental Health, Institute of Industrial Ecological Sciences, University of Occupational and Environmental Health, Japan, Fukuoka, 807-8555 Japan; 7 Department of Preventive Medicine and Public Health, Tokyo Medical University, Tokyo, 160-8402 Japan; 8 Department of Nursing, Japanese Red Cross College of Nursing, Tokyo, 150-0012 Japan

## Abstract

The effects of Pokémon GO, a new mobile game application which utilizes augmented reality, on risky behavior and health have already been discussed in anecdotal evidence. However, there have been no studies about its effects on mental health. This study investigated the relationships between Pokémon GO and psychological distress from an existing workers’ cohort in Japan. Online surveys were conducted to 3,915 full-time workers, at baseline (Nov 26, 2015–Feb 18, 2016) and at follow-up (Dec 1–4, 2016), using a self-report questionnaire. Pokémon GO players were defined as participants who had played Pokémon GO for one month or longer. Psychological distress was measured using validated scales. Of the completers, 246 (9.7%) had continued to play Pokémon GO. They were significantly younger than non-players. From the results of the general linear modeling, improvement in psychological distress was significantly greater among Pokémon GO players than among non-players (*p* = 0.025). Cohen’s *d* for the difference in psychological distress was −0.20 (95% CI, −0.33, −0.07). Pokémon GO may be effective for improving psychological distress among workers. Although its effect size is small, the game could have positive effects on the mental health of the adult working population.

## Introduction

Pokémon GO is a mobile application game released by Niantic, Inc. on July 2016, and was explosively downloaded (over 500 million) in just 8 weeks^1, 2^. Pokémon GO utilizes geographical data to create an augmented reality and enable players to search for, catch, and train Pokémon characters and obtain objects as if they were doing it in the real world. In addition, players can get their hands on “Pokémon eggs” in the game, and have to walk a certain distance to hatch them. Thus, in contrast to conservative video games, Pokémon GO requires players to go out of their homes or workplaces and move around to progress in the game.

Because of the novelty of this game’s concept, the negative and positive effects of Pokémon GO have been actively discussed in news and case reports, anecdotal evidence, and partially, in scientific studies. The potential negative effects of Pokémon GO could be traffic accidents or injury risks^3–6^; trespassing, abduction, and violence^7^; mosquito-borne diseases^8^; and sleep problems^9^. For instance, Wagner-Greene *et al* ^9^. conducted a cross-sectional survey among 662 adult Pokémon GO players; their findings indicated that more than a quarter of the players reported that they were likely to play Pokémon GO while driving and biking and they would also sacrifice sleep to play the game. Conversely, the potential merits of playing Pokémon GO have also been suggested, especially those on promoting physical activity^10–14^. In addition, Pokémon GO may help people with autism, depression, and social withdrawal (hikikomori) to move out of their home and thus, improve mental health^15–19^. These positive effects could be mediated by improved socialization^20, 21^, as well as by the increase in physical activity^22–24^. The technology of augmented reality offers data sharing and multi-user real-time interaction in the real world, which can satisfy the aspects of the sense of community: membership, influence, and integration and need fulfillment^21^. Moreover, since interventions with game-like elements to promote health can contribute to engaging individuals with various target populations and enhance their adherence to programs^25, 26^, Pokémon GO can help reach out to not only the motivated population, but also other populations (e.g., people not interested in health, people suffering from mental problems and having difficulties in accessing healthcare), regardless of their age or sex^13^.

However, most reports on the benefits of Pokémon GO on mental health were from case reports or anecdotal evidence^15–19^, and there is still no scientific evidence on this. In this study, we investigated the association between Pokémon GO and improved psychological distress, retrospectively, from an existing workers’ cohort in Japan. Mental health problems among workers are as common as community population^27, 28^, that are associated with poor quality of life and work performance^29, 30^. This is the first scientific study to report the association between playing Pokémon GO and mental health and can be useful for understanding the impact of the new smartphone game application on public health. We hypothesized that Pokémon GO would have a negative relationship with psychological distress.

## Methods

### Study design and setting

This study was a retrospective cohort study based on an online survey by an Internet survey company (Macromill, Inc.^31^). The company has access to more than 2,000,000 registered members of all prefectures in Japan. These registered members have diverse characteristics in terms of sex and age. Approximately half of them were women (56.3%), and their age ranged from 12 years to over 60 years. Regarding occupation, more than half (67.4%) of the members were workers (public servants [3.5%], employers [1.8%], workers employed by private companies [40.1%], freelancers [6.6%], part-time workers [15.4%]). Participants who met the eligibility criteria as described below were randomly sampled from the member pool and completed an online self-report questionnaire developed by the authors at baseline (Nov 26, 2015–Feb 18, 2016) and at follow-up (Dec 1–4, 2016). Pokémon GO was released on July 22, 2016 in Japan^1^, between the baseline and follow-up survey. This study protocol was received ethical approved by the research ethics committee of the Graduate School of Medicine and Faculty of Medicine, The University of Tokyo, Japan (No. 10856). The ethics committee confirmed that all methods in this study performed in accordance with the Ethical Guidelines for Medical and Human Research Involving Human Subjects, Japan^32^. We obtained informed consent from all participants via questionnaire instructions on the website. The instructions assured protection of personal information and explained that data would be anonymized. Any identifying information (participants’ name, and other identifiers that could lead to identification of a participant) was removed when we got the data through the Internet survey company, and no other images/videos were not obtained from the participants. Our study has been reported according to the Strengthening the Reporting of Observational studies in Epidemiology (STROBE) guidelines^33^.

### Participants

At baseline, a total of 3,915 workers who had registered with the Internet survey company were recruited and completed the survey in the order of arrival. The recruitment of participants was stratified by sex (males, females) and age (in years; 18–29, 30–39, 40–49, and 50 or more) among randomly sampled workers of the registered members. Participant inclusion criteria were (a) workers who lived in Japan; (b) workers employed by private companies, public servants, or freelancers; and (c) full-time workers. Participant exclusion criteria were (a) those not employed and (b) employed part-time workers. Based on these criteria, the Internet survey company randomly recruited workers from their potential pool of participants via e-mail and their own website. If the eligible workers agreed to the terms and conditions of the online survey, they could access the self-report questionnaire. Since the Internet survey company ceased recruitment once the target number of respondents had been reached, the response rate could not be determined. After about a year, the company recruited the workers to complete the baseline survey again via e-mail. The follow-up survey was managed by coordination staffs from the Internet survey company, sending several reminders to the participants who had not responded when first contacted. Participating workers were awarded approximately 80 “Macromill points” as a reward for participation in each survey, which could be used for cashing out and shopping (1 point was equivalent to JPY1).

### Variables and measurements

All variables were measured using the online self-report questionnaire that developed by the authors. The eligible workers who agreed to participate were instructed to log in to the portal and complete the online questionnaire. In the follow-up survey, we measured whether workers had played Pokémon GO. Primary (i.e., psychological distress) and secondary outcomes (i.e., physical complaints and work performance), covariates, and other demographic variables were measured at baseline and follow-up.

### Pokémon GO players

The exposure variable was measured with the dichotomous question, “During the past 1 year, have you continued to play Pokémon GO, which is a smartphone game app, for 1 month or longer”? Workers who answered “yes” to this question were considered Pokémon GO players.

### Psychological distress

Psychological distress was measured using the Brief Job Stress Questionnaire (BJSQ)^34^. It includes 18 items and 5 subscales: vigor (three items; e.g., “I have been very active”), irritation (three items; e.g., “I have felt angry”), fatigue (three items; e.g., “I have felt extremely tired”), anxiety (three items; e.g., “I have felt tense”), and depression (six items; “I have felt depressed”). All items were rated on a 4-point Likert scale (1: hardly; 4: almost). Since vigor measured a positive aspect of psychological distress, scores of vigor were reverse-coded when calculating the total scores. Higher scores indicated higher psychological distress. This scale has been widely used in Japan to assess responses to stress and has demonstrated satisfactory internal consistency, test-retest reliability, convergent validity, and predictive validity for the onset of depression^35, 36^.

### Physical complaints

Physical complaints were also measured using the BJSQ^34^, including 11 items and various types of self-reported complaints in the past 1 month: dizziness, arthralgia, headache, neck and shoulder pain, backache, eyestrain, aspiration, malfunction of stomach, loss of appetite, constipation and diarrhea, and insomnia (e.g., “I feel dizzy”). All items were rated on a 4-point Likert scale (1: hardly; 4: almost). Higher scores indicate higher physical complaints.

### Work performance

Work performance was assessed using an item from the validated scale, the Japanese short version of the WHO Health and Work Performance Questionnaire (WHO-HPQ)^37, 38^. The item rated an individual’s overall job performance for the past 1 month on a scale of 0 to 10 with 0 being the worst job performance and 10 being the best. According to a scoring rule of WHO-HPQ^39^, the ratings were multiplied by 10 to calculate work performance.

### Covariates and other demographic variables

As confounders, we measured sex, age, smoking habits (never, smoked previously but currently not smoking, and currently smoking), and drinking habits (never, rarely, sometimes, and daily drinking). Using the BJSQ, job stressors were also measured as confounders at the workplace^34^. Job stressors contained job demands (three items; e.g., “I have an extremely large amount of work to do”), job control (three items; e.g., “I can work at my own pace”), social support from supervisors (three items; e.g., “How freely can you talk with supervisors”?), and social support from colleagues (three items; e.g., “How reliable are colleagues when you are troubled”?). All items were rated on a 4-point Likert scale, and higher scores indicate higher job demands, job control, and social support from supervisors and colleagues. Marital status, educational status, employment status, job type, employment shift status, and household income were also measured as other demographic variables.

### Analyses

Descriptive statistics and Cronbach’s alphas for multi-item continuous scales (i.e., psychological distress, physical complaints, and job stressors) were calculated for each group. For the main analysis, general linear modeling for a two-way factorial Analysis of Variance (ANOVA) was performed. We estimated the fixed effects of group (Pokémon GO player/non-player), time (baseline/follow-up), and interaction (group*time) on psychological distress, physical complaints, and work performance. Sex, age, smoking habit, drinking habit, and job stressors at baseline were entered as covariates. Continuous covariates (i.e., age and job stressors) were mean-centered, and other covariates were transformed into dummy variables: sex (male [reference group]), smoking habits (never/previously smoking, but not currently [reference group]), and drinking habits (not/rarely [reference group]). The restricted maximum likelihood estimation (REML) was used for the estimation of parameters, and the MIXED model command of the PASW statistics version 18 (IBM SPSS software)^40^ was used for analyses. We calculated Cohen’s *d* ^41^ as effect sizes for each outcome, using descriptive means of change from baseline to follow-up and standard deviations between two groups. The effect size would be considered large, medium, and small if Cohen’s *d* had been 0.80, 0.50, and 0.20, respectively^41^. Since the study employed an online-based survey, there were no missing values for any of the variables or items. Further, since the exposure variable was measured after the baseline survey, attrition at follow-up could not be addressed. We only analyzed completed follow-up surveys.

## Results

### Characteristics of participants

Of the total of 3,915 workers at baseline, 2,599 workers completed the follow-up survey after Pokémon GO was released (valid follow-up rate = 66.4%). Due to employing the Internet survey via e-mail, reasons for no response (*N* = 1,316) were unknown to us. Sixty-nine of these participants had lost their jobs and were thus converted to non-workers and excluded from the analyses. Thus, a total of 2,530 workers (64.6%) were included in the analyses. Since the Internet-based survey required the participants to complete all the items, there were no missing values in any of the variables or items.

Among those who completed the follow-up survey, 246 (9.7%) had continued to play Pokémon GO for 1 month or longer (Table 1). These Pokémon GO players were significantly younger (*M* = 37.09 ± 10.85) than non-players (*M* = 42.66 ± 10.65). The other participant characteristics did not significantly differ between groups. Most participants were full-time (84.9%) and day-time shift (87.5%) workers.

**Table 1:** Characteristics of the participants at baseline (*N* = 2,530).

	Baseline (Nov 2015–Feb 2016)			
	Total *N* = 2,530	Pokémon GO players^‡^ *N* = 246 (9.7%)	Non-players *N* = 2,284 (90.3%)	*p* values for difference between groups
	*N* (%)	*N* (%)	*N* (%)	
Mean age (SD)	42.12 (10.79)	37.09 (10.85)	42.66 (10.65)	*p* < 0.001
Sex				*p* = 0.716
Male	1,580 (62.5)	151 (64.1)	1,429 (62.6)	
Female	950 (37.5)	95 (38.6)	855 (37.4)	
Marital status				*p* = 0.095
Unmarried	1,010 (39.9)	107 (43.5)	903 (39.5)	
Married	1,351 (53.4)	117 (47.6)	1,234 (54.0)	
Divorce/Widowed	169 (6.7)	22 (8.9)	147 (6.4)	
Educational status				*p* = 0.702
≤12 years	613 (24.2)	60 (24.4)	553 (24.2)	
13–16 years	1,731 (68.4)	171 (69.5)	1,560 (68.3)	
≥17 years	176 (7.0)	15 (6.1)	161 (7.0)	
Others	10 (0.4)	0 (0.0)	10 (0.4)	
Employment status				*p* = 0.188
Full-time	2,147 (84.9)	217 (88.2)	1,930 (84.5)	
Contract/Dispatched	351 (13.9)	25 (10.2)	326 (14.3)	
Others	32 (1.3)	4 (1.6)	28 (1.2)	
Job type				*p* = 0.101
Managerial	323 (12.8)	19 (7.7)	304 (13.3)	
Professional	394 (15.6)	42 (17.1)	352 (15.4)	
Technical	259 (10.2)	31 (12.6)	228 (10.0)	
Clerical	975 (38.5)	94 (38.2)	881 (38.6)	
Production/Skilled	383 (15.1)	44 (17.9)	339 (14.8)	
Others	196 (7.7)	16 (6.5)	180 (7.9)	
Employment shift status				*p* = 0.898
Day-time	2,215 (87.5)	216 (87.8)	1,999 (87.5)	
Shift-time/night-time	315 (12.5)	30 (12.2)	285 (12.5)	
Household income				*p* = 0.229
≤\2.99 million	357 (14.1)	42 (17.1)	315 (13.8)	
\3.00-\4.99 million	688 (27.2)	72 (29.3)	616 (27.0)	
\5.00-\7.99 million	676 (26.7)	63 (25.6)	613 (26.8)	
\8.00-\9.99 million	270 (10.7)	29 (11.8)	241 (10.6)	
\10.00-\14.99 million	193 (7.6)	19 (7.7)	174 (7.6)	
≥\15.00 million	50 (2.0)	2 (0.8)	48 (2.1)	
Unknown/Not answered	296 (11.7)	19 (7.7)	277 (12.1)	
Smoking				*p* = 0.067
Currently not smoking	1,939 (76.6)	177 (72.0)	1,762 (77.1)	
Currently smoking	591 (23.4)	69 (28.0)	522 (22.9)	
Drinking				*p* = 0.453
Not/rarely drinking	1,014 (40.1)	90 (36.6)	924 (40.5)	
Sometimes	928 (36.7)	98 (39.8)	830 (36.3)	
Everyday	588 (23.2)	58 (23.6)	530 (23.2)	

Note. ^‡^“Pokémon GO players” had continued to play Pokémon GO for 1 month or longer.

### Effects of playing Pokémon GO on outcomes

Table 2 shows the descriptive mean scores and Cronbach’s alphas for job stressors and outcomes. Cronbach’s alphas ranged from 0.72 to 0.94 in each survey, indicating enough internal consistency among all continuous variables. From the results of general linear modeling (Table 3), the interaction effect of group by time was significant on psychological distress (*p* = 0.025). Improvement of psychological distress was significantly greater among Pokémon GO players than among non-players. Improvements in physical complaints (*p* = 0.447) and work performance (*p* = 0.377) were not statistically significant. Although physical complaints among Pokémon GO players lessened to a slightly greater extent than among non-players, the difference was not significant. On the other hand, work performance decreased to a slightly greater extent, although not significantly so, among Pokémon GO players. Cohen’s *d* values of psychological distress, physical complaints, and work performance were −0.20, −0.07, and −0.07, respectively.

**Table 2:** Scores of job stressors, psychological distress, physical complaints, and work performance of the participants between two groups

	Range	Pokémon GO players^‡^ *N* = 246 (9.7%)		Non-players *N* = 2,284 (90.3%)		Cronbach’s α (*N* = 2,530)	
		Baseline	Follow-up	Baseline	Follow-up	Baseline	Follow-up
		*Mean (SD)*					
*Job stressors*							
Job demands	3–12	6.77 (2.29)	6.93 (2.17)	7.04 (2.15)	7.16 (2.23)	0.82	0.84
Job control	3–12	7.07 (2.15)	6.80 (1.89)	7.04 (1.90)	7.11 (1.90)	0.72	0.73
Social support from supervisors	3–12	8.23 (2.26)	8.46 (2.15)	8.21 (2.21)	8.30 (2.22)	0.82	0.83
Social support from colleagues	3–12	7.55 (2.14)	7.79 (2.08)	7.76 (2.12)	7.82 (2.14)	0.81	0.82
*Primary outcome*							
Psychological distress	18–72	42.95 (12.05)	40.81 (11.11)	40.28 (11.09)	40.30 (11.28)	0.94	0.94
*Secondary outcomes*							
Physical complaints	11–44	21.28 (7.24)	20.75 (6.72)	20.25 (6.98)	20.21 (7.29)	0.90	0.91
Work performance	0–100	59.11 (18.49)	57.28 (17.76)	58.17 (18.64)	57.82 (18.53)	—	

Note. ^‡^“Pokémon GO players” had continued to play Pokémon GO for 1 month or longer.

**Table 3:** Interaction effects of playing Pokémon GO by time on psychological distress, physical complaints, and work performance

	*Estimated mean (SE)* ^†^				Interaction effect	*p* value	Cohen’s *d* (95% CI)Δ (follow-up - baseline)
	Pokémon GO players^‡^		Non-players				
	Baseline	Follow-up	Baseline	Follow-up			
*Primary outcome*							
Psychological distress	42.00 (0.64)	39.86 (0.67)	40.38 (0.21)	40.40 (0.22)	*F* (1, 5035.26) = 5.03	0.025	−0.20 (−0.33, −0.07)
*Secondary outcomes*							
Physical complaints	20.85 (0.43)	20.32 (0.45)	20.29 (0.14)	20.26 (0.15)	*F* (1, 5038.32) = 0.58	0.447	−0.07 (−0.20, 0.07)
Work performance	59.70 (1.13)	57.87 (1.14)	58.11 (0.37)	57.76 (0.37)	*F* (1, 5046.82) = 0.78	0.377	−0.07 (−0.21, 0.06)

Note. ^‡^“Pokémon GO players” had continued to play Pokémon GO for 1 month or longer.

^†^Estimated mean scores were controlled by sex, age, smoking habit, and drinking habit, and job stressors.

## Discussion

The results supported our primary hypotheses, indicating significantly better improvement of psychological distress among Pokémon GO players compared to non-players. The quantitative findings are the first scientific suggestion for the effects of Pokémon GO, the new mobile application game utilizing augmented reality, on mental health among workers. The observed effect size for a decrease in psychological distress was small (*d* = −0.20). Pokémon GO might not have a visible impact on psychological distress on an individual-basis, but it could have a critical impact on public health, affecting the whole population, because of its tremendous popularity. Previous anecdotal evidence concerning its health benefits or epidemiological studies on the game’s impact on physical activity were limited to younger populations (e.g., children, adolescents, or persons 35 years or younger)^14, 15^. However, the present study suggests that working population may also enjoy the benefits of playing Pokémon GO, such as improved mental health. Our findings are in line with a previous study^13^ which reported that Pokémon GO could have health benefits for participants with different demographic characteristics (e.g., age, sex) because the negative association between playing Pokémon GO, and psychological distress remained after adjustment (sex, age, smoking and drinking habits, and job stressors). Therefore, playing games utilizing the technology of augmented reality could be recommended among workers with various characteristics.

How Pokémon GO and the technology of augmented reality improve psychological distress was not the focus of the present study, but this can be partially explained by the increased physical activity^18^ and socialization^9, 21^ among players. Pokémon GO and other social exergames (games that require physical activity to play)^41^ utilizing augmented reality entails Global Positioning System (GPS) that enables players to get real-time geographic feedback and be impressed by multiple players playing the game in the real world^20^. Therefore, they might become physically active, albeit unconsciously. In addition, this technology is installed in mobile applications, which is easy to play, has minimum involvement, and only requires going out with a small digital device. Moreover, in contrast to conservative video games, the following are the simple, required operations for playing these social exergames: tapping, swiping, and just moving. Therefore, respondents might be able to play these social exergames briefly to refresh and cope with their daily psychological distress. Other mechanisms, like social and multiplayer effects of the technology of augmented reality, may also influence physical and mental health^42^. Factors that possibly influence adherence to playing Pokémon GO or the effects of Pokémon GO on psychological distress (e.g., personal characters, stressful events not measured in the study, social interaction, and especially, physical activity) should be discussed in future studies, and a randomized controlled trial should be conducted for stronger evidence.

We did not observe significant improvement on physical complaints or work performance among Pokémon GO players. The effect of playing Pokémon GO could be limited to psychological distress only. Possible reasons for these results might be mental- and non-work-specific components of Pokémon GO: fun, pleasure, or entertainment. The previous meta-analysis indicated that game-based digital interventions could improve mental health, and suggested that entertainment-based games might be used for relaxation and pleasure, rather than changing behavioral or cognitive skills^43^. Therefore, Pokémon GO might be more strongly associated with psychological and emotional aspects of private lives than physical aspects and work among players.

Our study had several limitations. First, since we investigated the association retrospectively, a causal relationship cannot be inferred. Since participants were not exposed to Pokémon GO at baseline, we could not precisely capture the changes in outcomes by exposure. Second, we could only investigate the association between outcomes and playing Pokémon GO in the short term (1 month or longer). Previous studies^13, 14^ have indicated that an increase in physical activity was no longer observable after several months. The association in the long term could not be determined. Third, participants were not a random sample of workers but worked as monitors for the Internet survey company. Therefore, we cannot generalize our findings to all Japanese workers. Fourth, we could not calculate the valid response rate at baseline and could not determine reasons for non-participation at follow-up. In addition, the completion rate for the survey was not very high (66.4%). These limitations might have caused selection bias, overestimation of the associations between Pokémon GO and outcomes. Fifth, all the measurements were self-reported and therefore, may have caused information bias and measurement errors. The follow-up survey was conducted in December 2016, five months after the release of Pokémon GO. Thus, measurement of exposure might be distorted by recall bias. Finally, we should also consider regression to the mean, in view of higher baseline scores on psychological distress among Pokémon GO players, compared to non-players.

In conclusion, Pokémon GO may be effective for improving psychological distress among workers. Although its effect size was small, it could have a critical public health impact on the mental health of the adult working population. Further studies are needed to investigate the causal relationship, relationships to physical activity and social behaviors, and mechanisms to improve the mental health.
